# Purcell Effect and Beaming of Emission in Hybrid AlGaAs Nanowires with GaAs Quantum Dots

**DOI:** 10.3390/nano11112894

**Published:** 2021-10-29

**Authors:** Rodion R. Reznik, George E. Cirlin, Konstantin P. Kotlyar, Igor V. Ilkiv, Nika Akopian, Lorenzo Leandro, Valentin V. Nikolaev, Alexey V. Belonovski, Mikhail A. Kaliteevski

**Affiliations:** 1 Alferov University, Khlopina St. 8/3, 194021 St. Petersburg, Russia; george.cirlin@mail.ru (G.E.C.); konstantin21kt@gmail.com (K.P.K.); leha.s92.92@gmail.com (A.V.B.); m.kaliteevski@mail.ru (M.A.K.); 2 St. Petersburg State University, 13B Universitetskaya Emb, 198504 St. Petersburg, Russia; fiskerr@ymail.com; 3 ETU “LETI”, Professora Popova St. 5, 197376 St. Petersburg, Russia; 4 Department of Photonics Engineering, Technical University of Denmark, 2800 Kongens Lyngby, Denmark; nika.akopian@gmail.com (N.A.); lorenzo.leandro0@gmail.com (L.L.); 5 Ioffe Institute, Polytechnicheskaya St. 26, 194021 St. Petersburg, Russia; valia.nikolaev@gmail.com; 6 ITMO University, Kronverkskiy Pr. 49, 197101 St. Petersburg, Russia

**Keywords:** nanowire, quantum dot, directionality of emission, Purcell factor, waveguide modes, molecular beam epitaxy, micro photoluminescence

## Abstract

Control of directionality of emissions is an important task for the realization of novel nanophotonic devices based on nanowires. Most of the existing approaches providing high directionality of the light emitted from nanowires are based on the utilization of the tapered shape of nanowires, serving as nanoantenna coupling with the light waveguided in nanowire and the directional output beam. Here we report the beaming of the emitted light with wavelength near 800 nm by naturally formed core-shell AlGaAs NW with multiply GaAs quantum dots (QDs) diameter 30 nm and height 10 nm, while the diameter of NW 130 nm, what does not support efficient emission into waveguided modes, including the mode HE_11_. Experimental measurements show that intensity of emission for directions in the vicinity of the axis of NW is about two orders of magnitude higher than for perpendicular directions. The developed theoretical approach allowed us to calculate the probability of spontaneous emission for various directions and into waveguided modes and showed that highly directional radiation can be provided by the intrinsic emission properties of cylindrical NW. Our results suggest that for the small diameter of NW, directional emissions are associated with an TM_0_ leaky mode (when electric field oriented in axial direction) and therefore manifests in an existence of axial electric dipole transitions in quantum dots.

## 1. Introduction

Nanowires (NWs) with quantum dots (QDs) have been intensively studied due to their potential applications, especially as a source of quantum light [1,2,3]. In particular, it was shown that GaAs QDs in AlGaAs NWs are effective sources of single photons in the wavelength range 770–800 nm [4,5]. An important property of NW is its possibility to control spontaneous emission rates, providing an inhibition as well as an enhancement of spontaneous emissions (SE) [6,7,8]. Tunneling of the spontaneous emissions of QD in NW into a single photonic mode is a critically important task for the realization of practical devices and many efforts have been made to provide directional emission of radiation from NW with QD [9,10,11]. The common approach is based on an emission of QD into the fundamental waveguide mode HE_11_, and a subsequent beaming of radiation via tapering of the shape of NW [12,13].

A concentration of the attention on the HE_11_ mode (possessing non-zero radial component of the electric field near axis of the NW) is justified by the fact in GaAs quantum wells, and an electric dipole moment of the exciton ground state is oriented along the interface of quantum wells in the radial direction [7,10,14,15,16].

The ideal approach to the fabrication of nanowires is the “bottom-up” approach [1], in this case the grown nanowires are naturally aligned along the axis, which allows the emitter to interact in the best way with the modes of the waveguide. Authors of work [17] present a quantum-dot-in-nanowire system that reproducibly self-assembles in core–shell GaAs/AlGaAs nanowires. In this case, it is extremely difficult to control the size and location of quantum dots. In turn, using the technology of sequential growth of NWs and QDs, it is possible to control the number and position of perfectly aligned emitters coupled with the same optical mode. This provides an excellent platform for studying and applying nanowires.

However, in the case of NWs with QDs, due to technological features, it is not always possible to grow NWs with a diameter suitable for the efficient confinement of the fundamental waveguide mode HE_11_ (in the case of AlGaAs NWs with GaAs QDs, the NW diameter should be more than 180 nm [6]). At the same time, some experimental and theoretical results indicate that coupling of light and heavy holes and light hole excitons, structural peculiarities and strains could lead to the appearance of pronounced axial component of an electric dipole in NW [18,19,20,21,22,23]. Therefore, it seems timely to expand investigation of SE in NW beyond fundamental HE_11_ mode and analyze emission into TM_0_ mode as well, which has a non-zero axial component of electric field and unlike HE_11_ do not demonstrate suppression of SE for NW of small diameter.

**Figure 1:**
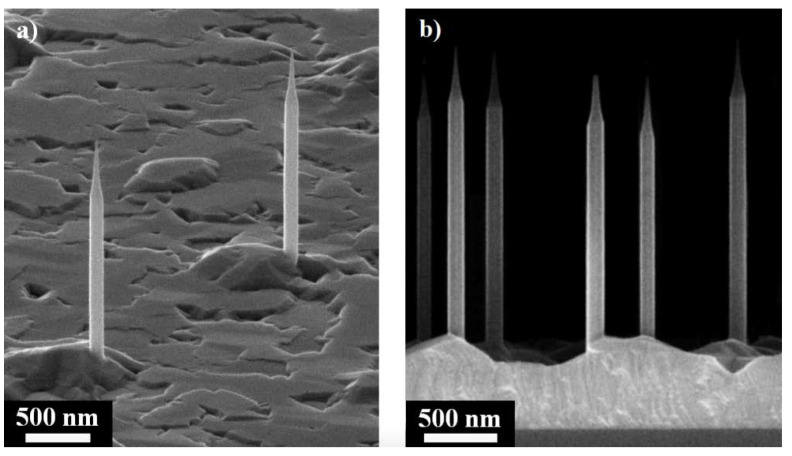

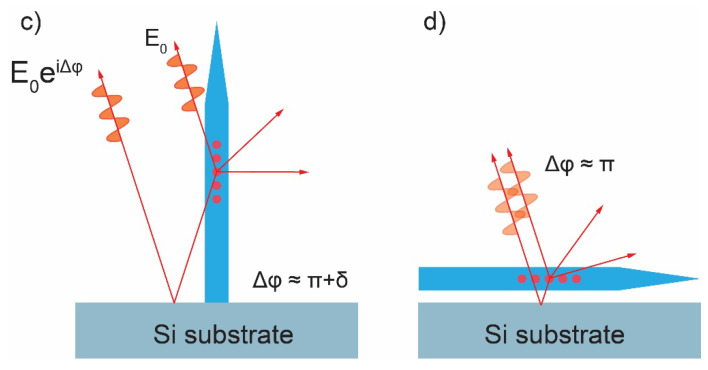
(**a**,**b**) SEM images of grown NWs on the substrate; (**c**,**d**) Schemes of the nanowire (shown) for standing vertically on a substrate (**c**), and lying on a substrate (**d**). The arrows illustrate the interference between the waves emitted by QD in the direction outside the substrate and reflected from the substrate.

In this paper, we present the results of experimental studies of directional radiation from GaAs QDs in AlGaAs NWs, where the NWs diameter is 130 nm (Figure 1), and the theoretical results describing the probability of spontaneous QD emissions for various directions. For such thin NW couplings of emissions, the fundamental waveguided mode HE_11_ is strongly suppressed [5].

## 2. Materials and Methods

Growth experiments were carried out using molecular-beam epitaxy (MBE) setup Riber Compact 21T (Riber, Bezons, France) equipped, in addition to a growth chamber, with a chamber for Au deposition (metallization chamber). Polished Si (111) wafers were used as substrates. The growth was carried out in several stages. The surface of wafers was treated in a 10:1 aqueous solution of HF, and then the samples were loaded to the metallization chamber and heated up to the temperature of 850 °C for 10 min. After that, the sample temperature was decreased to 550 °C, and gold film of ~0.2 nm thickness was deposited in the metallization chamber. To allow for the formation of Au droplets on the surface, the substrate temperature was maintained for one minute, and then the samples were cooled down to the room temperature and was transferred to the growth chamber without breaking the ultrahigh vacuum conditions. In the growth chamber, the substrate temperature was increased to 510 °C, and the Al, Ga and As shutters were opened after temperature stabilization for AlGaAs NWs growth under As-stabilized conditions. The reflection of high-energy electron diffraction (RHEED) patterns indicated the formation of a pure wurtzite crystallographic phase of NWs after 2 min of growth, which did not change during the entire process. A typical RHEED pattern after 5 min growth of AlGaAs NWs is shown in Figure S1. After 20 min of NW growth, GaA QDs were formed on top of the NWs by controllably closing the Al shutter for 15 s. Furthermore, the Al shutter was again opened for 45 s to form a cap layer. The QD formation procedure was repeated 10 times. At the final stage, the Al source shutter was opened for 5 min to form a cover layer. The nominal content of Al in the solid solution was set at 0.3 according to previous calibrations using a GaAs (100) substrate. The nominal growth rate of AlGaAs was kept constant at 1 monolayer per second (ML/s) during the whole experiment.

Morphological and structural properties of the grown samples were studied using a scanning electron microscope (SEM Supra 25, Carl Zeiss, Oberkochen, Germany) and a transmission electron microscope (TEM, JEOL 2100, JEOL Ltd., Tokyo, Japan) equipped with energy-dispersive X-ray spectroscopy analysis (EDX, X-Max 80, Oxford Ins., High Wycombe, UK). Optical properties of single NWs were examined using a micro-photoluminescence (μPL) setup (Princeton instruments, Trenton, NJ, USA) by a continuous-wave neodymium laser (wavelength 532 nm). A silicon-charged-coupled device (CCD) (Princeton instruments, Trenton, NJ, USA) was used as a photodetector. PL measurements were taken at a temperature of 4K in a closed cycle helium cryostat (Cryovac, Troisdorf, Germany). An optical microscope (Attocube, Haar, Germany) installed inside the cryostat allowed in situ control of the sample surface in the laser excitation region. Additionally, the setup is allowed to document macroPL from the array of NWs with QDs using a defocused laser beam. A typical spectrum from the array of AlGaAs NWs with GaAs QDs is shown in Figure S2.

## 3. Results

Typical SEM images of grown AlGaA NWs with GaAs QDs are shown in Figure 1a,b. It is seen that AlGaAs NWs have a pencil-like shape, with a diameter of 130 nm at the bottom and 20 nm at the top of NWs. The AlGaAs NWs formed 2 μm length strongly in the <111> direction, which indicates their epitaxial bond with the Si substrate (111). It is important to note that the surface density of NWs, corresponding to the density of gold droplets on the substrate surface, turned out to be 3 × 10^7^ cm^−2^. Such a density made it possible to excite a single NW on some areas of the substrate. In addition, a quasi-two-dimensional AlGaAs layer between the NWs on the surface of the substrate was observed.

According to our previous works, under the described growth conditions, AlGaA NWs are spontaneously formed by a core-shell structure [24]. Moreover, in the case of nominal Al_0.3_Ga_0.7_A composition, the real content of aluminum in the AlGaAs NW core is 16%, and 24% in the shell [25].

The GaAs QDs diameter is equal to the diameter of the AlGaAs NWs core [4], which, in turn, is determined by the size of the Au catalyst droplet, ~30 nm in our case [24]. Accordingly, in our case, the thickness of the NWs shell is ~50 nm. Knowing the growth time and height of the NWs, we can determine the heights of the GaAs QDs and the AlGaAs barrier between QDs, which are ~10.5 nm and ~45 nm, respectively. Examination by the EDX technique of the grown NWs confirmed the estimated values of both QDs and barriers (Figure S3).

To compare the intensity of emission from QDs in different directions, part of the NW was removed from the substrate surface and placed on the surface of a clean silicon wafer.

**Figure 2:**
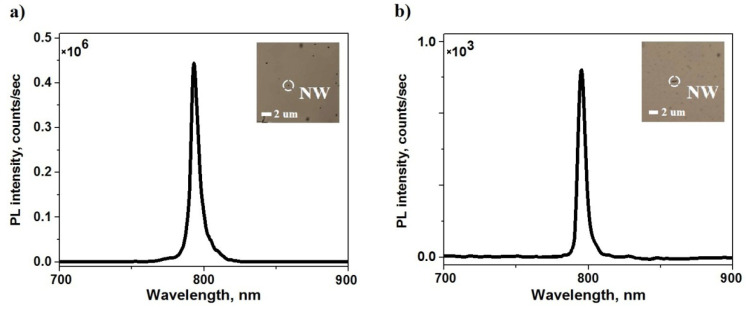
Spectra of microPL of the single NW with QD vertically standing on the substrate (**a**) as shown in Figure 1c, and lying on the substrate (**b**) as show in Figure 1d. The embedded pictures in (**a**,**b**) show the optical microscope images of the standing and lying single NW, respectively.

Figure 2 shows typical μPL spectra under identical conditions, corresponding to emission from GaAs QDs in single AlGaAs NWs grown in a direction perpendicular to the surface of the Si substrate (Figure 2a), as well as those dispersed onto the Si surface single NW (Figure 2b). In both cases, the PL signal from the QDs was collected in the direction perpendicular to the substrate surface.

The insertions in Figure 2a,b show the images of single standing and lying on the Si surface NW made with an optical microscope during the detection of μPL spectra. As can be seen from the figures, the wavelengths of the μPL spectra peaks are almost identical in both cases and amount to ~795 nm. However, the μPL intensity of lying on the surface nanostructure is ~2 orders of magnitude lower than that of not removed from the substrate surface. It is important to note that in the absence of the NWs within the laser beam, the μPL signal was not observed. This fact excludes the influence of the quasi-two-dimensional layer formed during the synthesis of NWs on the obtained μPL spectra. All of the above indicates that the μPL emission from GaAs QDs in AlGaAs NWs follows the direction of the NW growth. In Figure S4 we present PL spectra taken from different standing and lying NWs showing the same trend.

## 4. Theory

The formalism used for the calculation is cumbersome and therefore is presented in the Supplementary Information (Section S2).

There are only two types of cylindrical waveguide modes with a non-zero electric field at the NW axis: TM-polarized modes with the azimuthal number m = 0 and HE/EH modes with m = ±1. TM modes have an electric field parallel to the NW axis (axial filed direction), whereas modes with m = ±1, which include the fundamental HE_11_ mode, demonstrate an electric field perpendicular to the structure axis (radial field direction). The leaky modes responsible for direct radiation into outer space have the same electric field direction on the axis (see Supplementary Information (Section S2)). In the local response approximation, the quantum dots in the center of NW will radiate only into the aforementioned modes. The intensity of spontaneous emission into these modes is proportional to the square amplitude of axial (for TM modes) or radial (for |m| = 1 modes) components of the dipole matrix element of the ground optical transition in quantum dots under study.

**Figure 3:**
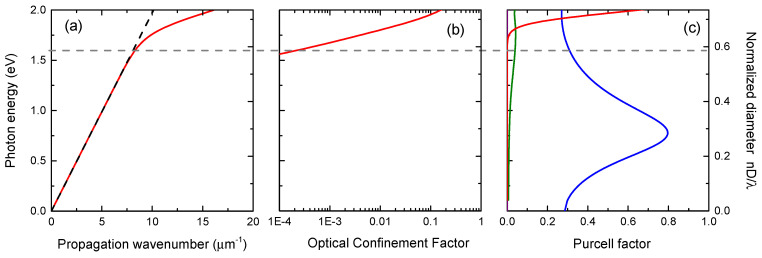
(**a**) Dispersion for HE11 mode in NW; (**b**) optical confinement factor g for HE_11_ mode; (**c**) Purcell factor for dipole on the axis interacting with NW for HE_11_ mode (red line), leaky modes interacting with radial dipole (green line) and axial dipole interacting with TM_0_ leaky modes (blue line).

Figure 3a demonstrates the dispersion dependence for the HE_11_ mode in NW with a diameter 130 nm. It can be seen that formally the waveguided mode exists for any photon energy, but below 1.5 eV it coincides with the light cone for the air (media outside NW). In Figure 3b, a calculation of the optical confinement factor g of the HE_11_ mode is presented, which is defined as a relation of the electromagnetic field energy inside NW to the total energy in the mode. One may see that in the low energy/diameter region where the HE_11_ dispersion curve approaches the light cone, factor g plunges to extremely small values. This means that despite formal existence of the waveguided mode HE_11_ in that region, the electromagnetic field is delocalized and the interaction of a quantum dot inside NW with the fundamental HE_11_ mode is weak. For the photon energy 1.55 eV corresponding to the emission of QD (shown by horizontal dashed line in Figure 3), the optical confinement factor g is about 8 × 10^−5^.

It is convenient to describe the modification of emission efficiency by Purcell factors defined as spontaneous emission rates into particular channels divided by an emission rate of a QD in homogenous bulk material. Purcell factors for spontaneous emission of a radial-dipole optical transition into HE_11_ mode (red curve) and into leaky modes (green curve) are shown in Figure 3c alongside a total Purcell factor for axial-dipole transition (blue curve), which in the considered range of photon energies or diameters coincides with the Purcell factor for TM-polarized leaky modes (see Supplementary Information (Section S2)). It can be seen that for the diameter of NW 130 nm and photon energy 1.55 eV emission into waveguided mode HE_11_ is suppressed: the value of the Purcell factor is about 5.6 × 10^−5^. On the other hand, for the leaky modes interacting with a radial–dipole transition in the QD Purcell factor has the value 3.7 × 10^−2^, which exceeds the corresponding value for the waveguided mode HE_11_ by three orders of magnitude.

**Figure 4:**
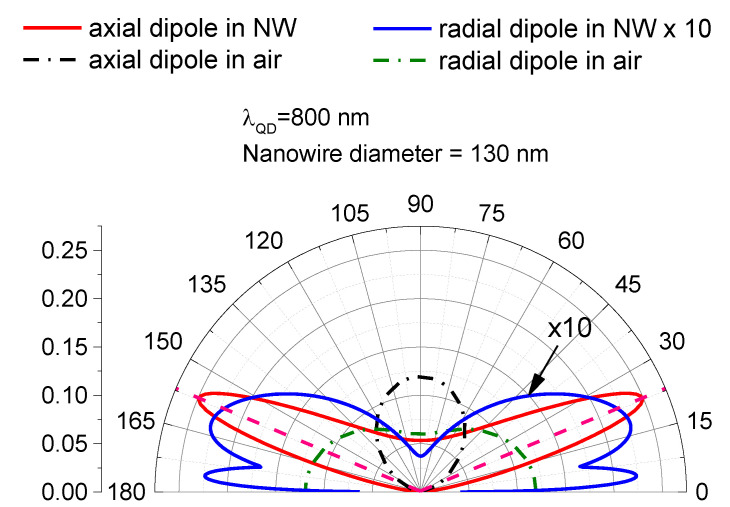
Directionality diagrams for the probability of emission for the photon energy 800 nm for the emission of axial dipole (red line) and for radial dipole (blue line). For radial directionally, the diagram is averaged by rotating it around an axis of NW. Dashed black and green lines show the emission of an axial dipole and for the radial dipole in air, respectively. The axis of the nanowire corresponds to the direction 0–180 degrees.

It interesting to analyze how NW modifies the directionality of the emitted radiation. The blue line in Figure 4 shows the directionality diagram for a radial–dipole optical transition (in this case an emitter placed on the NW axis radiates through HE/EH leaky modes with c azimuthal number |m| = 1). It can be seen that radiation is confined within the cone around the NW axis with the angle at approximately 45 degrees, where it is distributed more or less uniformly. Such peculiar directionality distribution can qualitatively explain the observed effect of the “axial” channeling of emitted radiation.

For TM-leaky modes, the value of the Purcell factor exceeds those for |m| = 1 leaky modes by one order of magnitude (0.32 vs. 3.7 × 10^−2^), as shown in Figure 3c.

Resent observations of emissions from QD due to optical transitions, which have an axial–dipole component, require us to pay attention to emissions via TM leaky modes. This is especially important in relation to structures considered in this work since GaAs QDs have a relatively high aspect ratio and the stress distribution in core-shell QDs embedded in a nano-wire might be qualitatively different from the case of conventional QDs. Figure 4 shows a directionality diagram for TM leaky modes. It can be seen that for TM leaky modes, emissions are sharply channeled at the directions which form angle 22 degrees with the axis of NW, and the width of the beam is about 10 degrees. Such sharp channeling could be helpful for the manipulation of the emitted photons, which is important for the development of various quantum light devices.

In experiments, radiation emitted by quantum dots in vertically standing NWs or NWs on the substrate is collected by the objective and is characterized by certain numerical apertures or cone angles. Qualitative analysis shows (see Section S3 of Supplementary Materials) that for the vertically standing NW, radiation power collected by an objective should be almost one order of magnitude larger than for the lying one (see Figure S5 of Supplementary Materials).

Finally, we should note that there is a discrepancy between an experimentally observed two order of magnitude difference of intensities of emitted radiation in “axial” and “radial” directions of emission. It can be seen from Figure 4 that the difference of intensities of emission in “axial” and radial directions do not exceed one order of magnitude.

To explain two orders of magnitude differences in intensity, one should seek for additional reasons. One possible explanation could be based on negative interference of the wave, emitted in particular in the radial direction, and toward the substrate, and is then reflected from the substrate in the direction of the first wave. Reflection from the substrate gives the phase a shift near p, and two waves weaken each other, as shown in Figure 1d. On other hand, the vertical alignment of NW, the phase shift between the wave emitted upward, and the wave emitted toward the substrate and reflected from it are about the same, and the two waves do not experience a negative interference. A qualitative numerical analysis of these effects is provided is Supplementary Materials Section S3. It can be seen that the direction-dependent modification of the emission probability together with the negative interference (in the case of lying NW) of the waves emitted by QD outward from the substrate, emitted toward the substrate and reflected back (see Figure S6 of Supplementary Materials), can explain the observed differences in the emission intensity.

## 5. Conclusions

A set of core-shell AlGaA nanowires multiplied by GaAs QDs has been grown using molecular beam epitaxy. The diameter of NW was about 130 nm, when the fundamental mode HE_11_ was screened and other waveguided modes do not exist. Each QD has a height of 10.5 nm and a diameter of 30 nm. The micro photoluminescence of NW was measured in the direction along the axis of the NW and in the perpendicular direction. QDs demonstrate the emission lines with wavelengths near 800 nm, and an intensity of emission in the direction around the axis of NW is about two orders of magnitude larger than that for the perpendicular direction. The probabilities of spontaneous emissions for various directions and Purcell factors were calculated. It was shown that for TM leaky modes (interacting with an axial electric dipole), the probability of emission is more than one order of magnitude larger than the joint probability of emission for all leaky modes and fundamental HE11 modes interacting with a radial dipole. Our results suggest that: (i) highly directional emissions can be obtained from NW without the utilization of a waveguiding effect; and (ii) ground optical transition of GaAs QDs in AlGaAs NW might possess a significant component of electric dipole matrix elements along the NW axis.

## Data Availability

The data presented in this study are available on request from the corresponding author. The data are not publicly available due to privacy.

## Supplementary Materials

The following are available online at https://www.mdpi.com/article/10.3390/nano11112894/s1, Figure S1: Typical RHEED pattern after 5 minutes of AlGaA NWs Growth; Figure S2: Typical macro-PL spectrum at 4K of AlGaA NWs with GaAs QDs; Figure S3: Typical EDX spectrum of AlGaAs NW with GaAs QDs; Figure S4: Typical spectra of microPL of the single NWs with QDs vertically standing on the substrate (a) and lying on the substrate (b); Figure S5: (a) Ratio of integrated emission probability inside the cone oriented along axis of NW and inside the cone oriented in perpendicular direction for axial orientation of dipole (blue) and radial orientation (red) as a function of the cone angle; (b) The scheme illustrating calculation of the Directional parameter; Figure S6: Distribution of the time-averaged Poynting vector around the NW, standing vertically on a Si substrate (a) and lying on a Si substrate (b).
