# Supplementary material for: Purcell Effect and Beaming of Emission in Hybrid AlGaAs Nanowires with GaAs Quantum Dots
Source: Nanomaterials (Basel). 2021 Oct 29;11(11):2894. doi: 10.3390/nano11112894 (PMC8617613; doi:10.3390/nano11112894)
Supplement: Supplementary file 1 [file nanomaterials-11-02894-s001.zip › nanomaterials-1421369-supplementary.pdf]

nanomaterials

Supplementary Information

Purcell Effect and Beaming of Emission in Hybrid AlGaAs Nanowires with GaAs Quantum Dots

Rodion R. Reznik [1,2,*], George E. Cirlin [1,3], Konstantin P. Kotlyar [1], Igor V. Ilkiv [2], Nika Akopian [4], Lorenzo Leandro [4], Valentin V. Nikolaev [5], Alexey V. Belonovski [1,6] and Mikhail A. Kaliteevski [1,6]

[1] Alferov University, Khlopina St. 8/3, St. Petersburg 194021, Russia; george.cirlin@mail.ru (G.E.C.); konstantin21kt@gmail.com (K.P.K.); leha.s92.92@gmail.com (A.V.B.); m.kaliteevski@mail.ru (M.A.K.)
[2] St. Petersburg State University, 13B Universitetskaya Emb, St. Petersburg 198504, Russia; fiskerr@ymail.com
[3] ETU "LETI", Professora Popova St. 5, St. Petersburg 197376, Russia
[4] Department of Photonics Engineering, Technical University of Denmark, 2800 Kongens Lyngby, Denmark; nika.akopian@gmail.com (N.A.); lorenzo.leandro0@gmail.com (L.L.)
[5] Ioffe Institute, Polytechnicheskaya St. 26, St. Petersburg 194021, Russia; valia.nikolaev@gmail.com
[6] ITMO University, Kronverkskiy Pr. 49, St. Petersburg 197101, Russia
* Correspondence: moment92@mail.ru

Section S1. Additional Experimental Data.

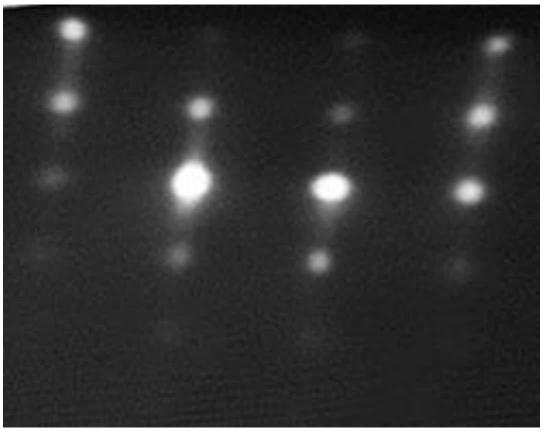

Figure S1. Typical RHEED pattern after 5 minutes of AlGaA NWs Growth.

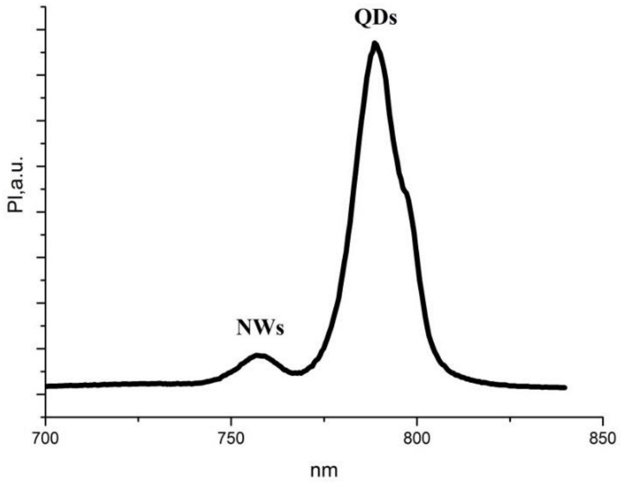

Figure S2. Typical macro-PL spectrum at 4K of AlGaA NWs with GaAs QDs.

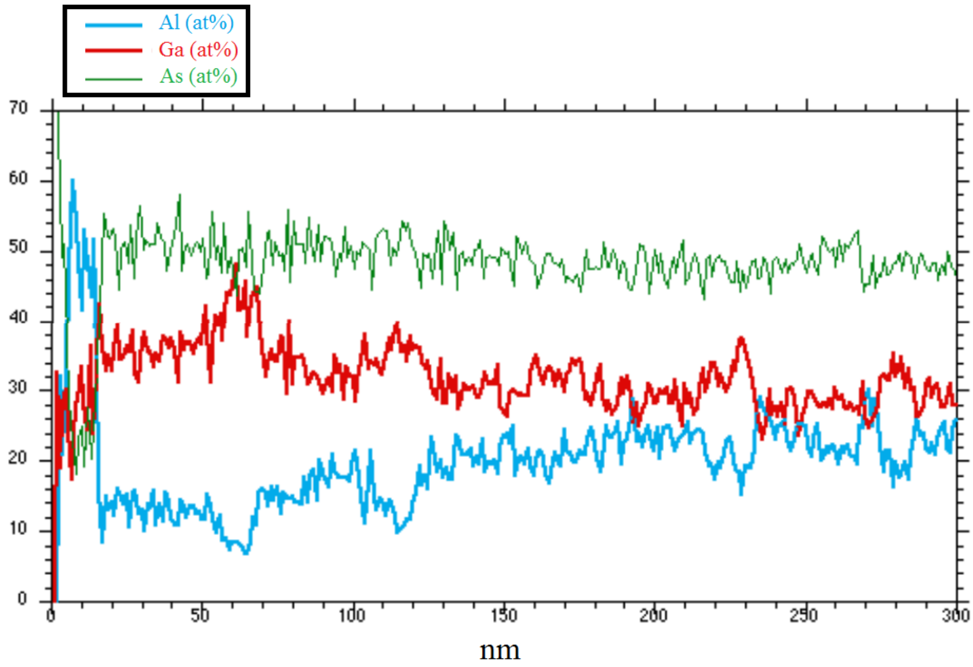

Figure S3. Typical EDX spectrum of AlGaAs NW with GaAs QDs.

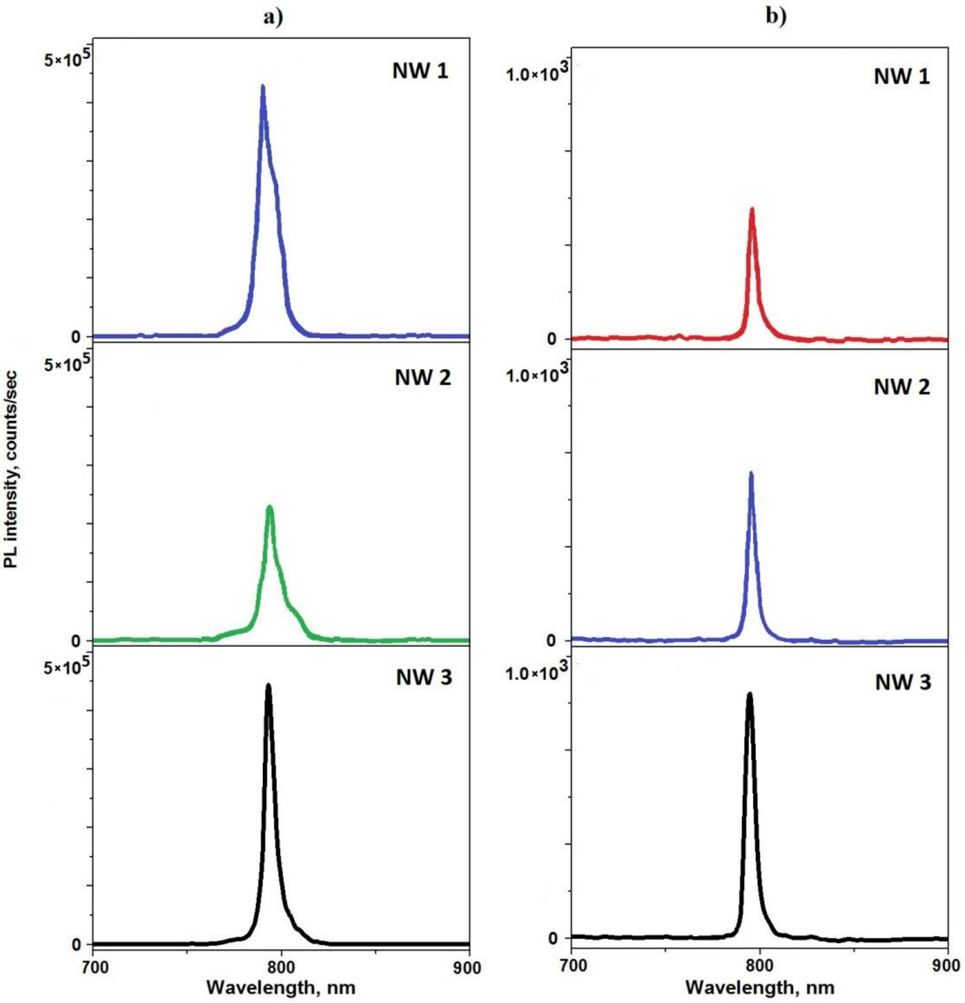

Figure S4. Typical spectra of microPL of the single NWs with QDs vertically standing on the substrate (**a**) and lying on the substrate (**b**).

Section S2. Purcell Factor for Waveguide and Leaky Modes.

a) Purcell Factor for Waveguide Modes

The dispersion equation for an infinite non-magnetic cylindrical waveguide with the radius ρ_0 and dielectric permittivity ε in air is:

$$\left(\frac{k_0}{|k_{\rho1}|}\frac{K'_m\left(|k_{\rho1}|\rho_0\right)}{K_m\left(|k_{\rho1}|\rho_0\right)}+\varepsilon\frac{k_0}{k_{\rho2}}\frac{J'_m\left(k_{\rho2}\rho_0\right)}{J_m\left(k_{\rho2}\rho_0\right)}\right)\left(\frac{k_0}{|k_{\rho1}|}\frac{K'_m\left(|k_{\rho1}|\rho_0\right)}{K_m\left(|k_{\rho1}|\rho_0\right)}+\frac{k_0}{k_{\rho2}}\frac{J'_m\left(k_{\rho2}\rho_0\right)}{J_m\left(k_{\rho2}\rho_0\right)}\right)=\left(\frac{k_z k_{\phi m}}{k_{\rho1}k_{\rho2}}\right)^2\left(\frac{k_{\rho1}}{k_{\rho2}}-\frac{k_{\rho2}}{k_{\rho1}}\right)^2 \quad (1)$$

Here $k_0 = \omega / c$ is a free-space light wavevector; $k_{\phi m} = m / \rho_0$ where m is an integer cyclic number; J_m and K_m are the Bessel functions of the first kind and the modified Bessel functions respectively; and $|k_{\rho1}| = \sqrt{k_z^2 - k_0^2}$ ($k_{\rho2} = \sqrt{\varepsilon k_0^2 - k_z^2}$) is the transverse component of the wavevector in air (in the waveguide material) which depends on the propagation wavenumber k_z Solving equation (1) we obtain dispersion dependencies $\omega^c_{m,\nu}\left(k_z\right)$ for different waveguide modes and are able to calculate the mode group velocities:

$$v^g_{m,\nu} = \frac{d\omega^c_{m,\nu}}{dk_z} \quad (2)$$

We define the Purcell factor as a ratio of the spontaneous emission rate into a particular channel to the total spontaneous emission rate of a dipole emitter in the bulk material with dielectric permittivity ε. In order to find the expression for the spontaneous emission rate into a waveguide mode we use 1. Fermi's Golden Rule 2. standard Born–von Karman periodic boundary conditions on the top and bottom facets of a quantization cylinder [1] 3. normalization of the waveguide mode to the single photon energy. As a result we obtain a simple dimensionless expression for the Purcell factor for the waveguide-mode luminescence:

$$F_{WG} = \frac{3}{8}\frac{1}{\sqrt{\varepsilon}}\frac{c}{v^g_{m,\nu}}\frac{\left|\mathbf{E}^{m,\nu}_{WG}\cdot\mathbf{e}_d\right|^2}{k_0^2 \times S_{m,\nu}} \tag{3}$$

Here $\mathbf{e}_d$ is the unit vector which defines polarization of the dipole optical transition; $\mathbf{E}^{m,\nu}_{WG}$ is the (complex) electric field profile of the waveguide mode at the emitter position and $S_{m,\nu}$ is the energy normalization integral:

$$S_{m,\nu} = 2\pi\int_0^{+\infty} p_{m,\nu}(\rho)\rho d\rho$$
$$p_{m,\nu}(\rho) = \frac{1}{16\pi}\left(\varepsilon(\rho)\left|\mathbf{E}^{m,\nu}_{WG}(\rho)\right|^2 + \left|\mathbf{H}^{m,\nu}_{WG}(\rho)\right|^2\right) \tag{4}$$

where ρ is the distance from the waveguide axis; $\mathbf{H}^{m,\nu}_{WG}$ is the magnetic-field profile of the mode and $p_{m,\nu}(\rho)$ is the density of electromagnetic energy (here we use the Gaussian unit system). The expression (3) is qualitatively similar to the previously used variants [1,2] but with important difference that Eq. (4) is universal and does account for the cylindrical character of the electromagnetic field in the waveguide whereas in Refs [1,2] implicit assumption of equality of electric and magnetic parts of energy is used which is true only for plain waves. We note that the scaling of electromagnetic field of the waveguide mode in Eq.(3,4) is arbitrary; it is sufficient to use the same electromagnetic profile in Eqs. (3) and (4).

The modes with $m = \pm 1$ have only the radial nonzero component on the waveguide axis:

$$\mathbf{E}^{m=\pm1,\nu}_{WG}(\rho = 0) \propto \mathbf{e}_x \pm i\mathbf{e}_y \tag{5}$$

where $\mathbf{e}_{x,y}$ are the Cartesian basis vectors perpendicular to the waveguide axis. Therefore, the fundamental waveguide mode HE_{11} interacts only with the dipole transitions on the waveguide axis which have nonzero radial component of the dipole matrix element: $(\mathbf{e}_x \pm i\mathbf{e}_y)\times\mathbf{e}_d \neq 0$.

b) Purcell Factor for Leaky Modes

The electromagnetic field in the external medium can be written as a sum of TM-polarized waves

$$E_z^{TM} = \frac{1}{2}\frac{k_{\rho 1}}{k_0}\left(A_{TM}^{+}H_m^{(1)}\left(k_{\rho 1}\rho\right) + A_{TM}^{-}H_m^{(2)}\left(k_{\rho 1}\rho\right)\right) \qquad E_\phi^{TM} = -\frac{1}{2}\frac{k_z k_{\phi m}}{k_0 k_{\rho 1}}\left(A_{TM}^{+}H_m^{(1)}\left(k_{\rho 1}\rho\right) + A_{TM}^{-}H_m^{(2)}\left(k_{\rho 1}\rho\right)\right)$$

$$E_\rho^{TM} = i\frac{1}{2}\frac{k_z}{k_0}\left(A_{TM}^{+}H_m'^{(1)}\left(k_{\rho 1}\rho\right) + A_{TM}^{-}H_m'^{(2)}\left(k_{\rho 1}\rho\right)\right) \qquad H_\phi^{TM} = i\frac{1}{2}\left(A_{TM}^{+}H_m'^{(1)}\left(k_{\rho 1}\rho\right) + A_{TM}^{-}H_m'^{(2)}\left(k_{\rho 1}\rho\right)\right) \tag{6}$$

$$H_\rho^{TM} = \frac{1}{2}\frac{k_{\phi m}}{k_{\rho 1}}\left(A_{TM}^{+}H_m^{(1)}\left(k_{\rho 1}\rho\right) + A_{TM}^{-}H_m^{(2)}\left(k_{\rho 1}\rho\right)\right)$$

$$k_{\rho 1} = \sqrt{\frac{\omega^2}{c^2} - k_z^2}$$

and TE-polarized waves

$$E_\phi^{TE} = -i\frac{1}{2}\left(A_{TE}^{+}H_m'^{(1)}\left(k_{\rho 1}\rho\right) + A_{TE}^{-}H_m'^{(2)}\left(k_{\rho 1}\rho\right)\right) \qquad E_\rho^{TE} = -\frac{1}{2}\frac{k_{\phi m}}{k_\rho}\left(A_{TE}^{+}H_m^{(1)}\left(k_{\rho 1}\rho\right) + A_{TE}^{-}H_m^{(2)}\left(k_{\rho 1}\rho\right)\right)$$

$$H_z^{TE} = \frac{1}{2}\frac{k_\rho}{k_0}\left(A_{TE}^{+}H_m^{(1)}\left(k_{\rho 1}\rho\right) + A_{TE}^{-}H_m^{(2)}\left(k_{\rho 1}\rho\right)\right) \quad H_\phi^{TE} = -\frac{1}{2}\frac{k_z k_{\phi m}}{k_0 k_\rho}\left(A_{TE}^{+}H_m^{(1)}\left(k_{\rho 1}\rho\right) + A_{TE}^{-}H_m^{(2)}\left(k_{\rho 1}\rho\right)\right) \tag{7}$$

$$H_\rho^{TE} = i\frac{1}{2}\frac{k_z}{k_0}\left(A_{TE}^{+}H_m'^{(1)}\left(k_{\rho 1}\rho\right) + A_{TE}^{-}H_m'^{(2)}\left(k_{\rho 1}\rho\right)\right)$$

Here the plus and minus superscripts at A coefficients show the direction of wave propagation from and to the structure axis (the time dependence of the form $\exp\left(-i\omega t\right)$ is presumed); $H_m^{(1,2)}$ are Hankel functions.

Unlike the waveguide modes the leaky radiative modes which transfer energy directly into external medium are not localized in the vicinity of the structure. Therefore, the quantization procedure should involve not only the top and bottom facets of the quantization cylinder but the cylindrical surface as well. Here we generalize the approach of Ref. [3]: the tangential components of electric $\mathbf{E}$ and magnetic $\mathbf{H}$ fields are required to be equal to zero on the cylindrical surface of the quantization cylinder and then the geometric dimensions (the length L_z and the radius R) of the cylinder are put to infinity. Using Fermi's Golden rule and the normalization of the electromagnetic field energy contained in the quantization cylinder to the energy of a single photon we derive the Purcell factor of the leaky modes. The luminescence of the quantum emitter on the waveguide axis can be described by the following Purcell factor

$$F_L = \frac{3}{32\pi^2}\frac{1}{\sqrt{\varepsilon}}\frac{c}{\omega}\sum_{m=0,\pm 1}\sum_{J=EH,HE}\int_0^\pi \frac{\left|\mathbf{E}_J^m\left(\rho=0\right)\cdot\mathbf{e}_d\right|^2}{\rho_{J\,E2D}^m}d\theta$$

$$\rho_{J\,E2D}^m = \frac{W_{EM}}{2\pi R L_z} = \lim_{R\to\infty}\frac{1}{R}\int_0^R p_J^m\left(\rho\right)\rho d\rho \tag{8}$$

$$p_J^m\left(\rho\right) = \frac{1}{16\pi}\left(\varepsilon\left(\rho\right)\left|\mathbf{E}_J^m\left(\rho\right)\right|^2 + \left|\mathbf{H}_J^m\left(\rho\right)\right|^2\right)$$

Here θ is the angle between the structure axis and the leaky mode propagation direction and $\rho_{J\,E2D}^m$ is the total electromagnetic energy W_{EM} enclosed inside the quantization cylinder reduced to the arear of the cylindrical surface $2\pi R L_z$. If the electromagnetic field in the external space is a sum of cylindrical waves, Eqs. (6, 7), the limit of $\rho_{J\,E2D}^m$ for the infinite radius R is finite and well defined:

$$\rho_{E2D} = \frac{1}{8\pi^2 k_{\rho 1}}\left\{\left|A_{TM}^{-}\right|^2 + \left|A_{TE}^{-}\right|^2\right\} \tag{9}$$

Deriving (9) we presumed that light absorption is negligible.

To be able to use equation (8) one needs to find the electric field $\mathbf{E}_J^m\left(\rho=0\right)$ of the leaky mode at the structure axis. Using the aforementioned boundary conditions, we found that the leaky modes can be divided into two types of mixed modes: EH-like and HE-like modes, similarly to the case of the waveguide modes. For the EH-like modes the amplitude coefficient A_{TE}^- of the convergent TE-polarized wave is proportional to the coefficient A_{TM}^- of the convergent TM-wave:

$$A_{TE}^- = -\frac{\tilde{r}_{TM\to TE}}{\frac{1}{2}\left(\tilde{r}_{TM\to TM}+\tilde{r}_{TE\to TE}\right)+\tilde{D}}A_{TM}^-$$
$$\tilde{D}=\left\{\frac{1}{4}\left(\tilde{r}_{TM\to TM}+\tilde{r}_{TE\to TE}\right)^2-\tilde{r}_{TE\to TM}\tilde{r}_{TM\to TE}\right\}^{\frac{1}{2}} \tag{10}$$

and for HE leaky modes the relation is

$$A_{TM}^- = -\frac{\tilde{r}_{TE\to TM}}{\frac{1}{2}\left(\tilde{r}_{TM\to TM}+\tilde{r}_{TE\to TE}\right)+\tilde{D}}A_{TE}^- \tag{11}$$

In Eqs. (10,11) the reflection coefficients $\tilde{r}$ are introduced as ratios of the amplitude coefficients A^+ of the diverging waves of curtain polarizations to the amplitude coefficient of the incident convergent wave A^- (hence the problem of incidence and reflection of cylindrical waves for the homogeneous waveguide is considered). These coefficients can be written in the form of a matrix:

$$\hat{s}=\begin{pmatrix}\tilde{r}_{TM\to TM} & \tilde{r}_{TE\to TM}\\ \tilde{r}_{TM\to TE} & \tilde{r}_{TE\to TE}\end{pmatrix}=\begin{pmatrix}\dfrac{A_{TM}^+}{A_{TM}^-} & \dfrac{A_{TM}^+}{A_{TE}^-}\\ \dfrac{A_{TE}^+}{A_{TM}^-} & \dfrac{A_{TE}^+}{A_{TE}^-}\end{pmatrix} \tag{12}$$

We found the explicit form of these coefficients for a homogeneous waveguide:

$$\hat{s}=-\frac{1}{\left(r_{TM}^{ax}Z_p^{11}+Z_p^{12}\right)\left(r_{TM}^{ax}Z_m^{11}+Z_m^{12}\right)-U^2\left(1+r_{TM}^{ax}\right)^2}\frac{1}{r_1^{ax}}\times$$
$$\begin{pmatrix}\left(r_2^{ax}Z_p^{21}+Z_p^{22}\right)\left(r_{TM}^{ax}Z_m^{11}+Z_m^{12}\right)-U^2\left(1+r_2^{ax}\right)^2 & -iU_{1\to2}\left(\tilde{C}_1^{(1)}-\tilde{C}_1^{(2)}\right)\left(1+r_2^{ax}\right)^2\\ iU_{1\to2}\left(\tilde{C}_1^{(1)}-\tilde{C}_1^{(2)}\right)\left(1+r_2^{ax}\right)^2 & \left(r_2^{ax}Z_p^{11}+Z_p^{12}\right)\left(r_2^{ax}Z_m^{21}+Z_m^{22}\right)-U^2\left(1+r_2^{ax}\right)^2\end{pmatrix}$$
$$Z_p^{ij}=Y_1\tilde{C}_1^{(i)}-Y_2\tilde{C}_2^{(j)} \qquad Z_m^{ij}=Y_1^{-1}\tilde{C}_1^{(i)}-Y_2^{-1}\tilde{C}_2^{(j)} \tag{13}$$
$$\tilde{C}_i^{(1,2)}=\frac{K}{k_{\rho i}}\frac{H_m'^{(1,2)}\left(k_{\rho i}\rho_0\right)}{H_m^{(1,2)}\left(k_{\rho i}\rho_0\right)} \qquad r_i^{ax}=\frac{H_m^{(1)}\left(k_{\rho i}\rho_0\right)}{H_m^{(2)}\left(k_{\rho i}\rho_0\right)}$$
$$Y=\sqrt{\frac{\varepsilon}{\mu}} \qquad U=\frac{k_z k_{\phi m}}{k_{\rho 1}k_{\rho 2}}\left(\frac{k_{\rho 1}}{k_{\rho 2}}-\frac{k_{\rho 2}}{k_{\rho 1}}\right)=\frac{m}{\rho_0}k_z\left(\frac{1}{k_{\rho 2}^2}-\frac{1}{k_{\rho 1}^2}\right)$$

In (13) subscript 1 designate parameters of the external medium and subscript 2 stands for the parameters of waveguide material. Using Eqs (6,7,10,11,13) one can find the electromagnetic field of the quantized leaky mode in the external medium. The field is proportional to the arbitrary coefficient A_{TM}^- for EH-leaky modes and to A_{TE}^- for HE-leaky modes. Using continuity of the tangential field components on the cylindrical interface of the waveguide one can find the electric field in the center of the structure

which is proportional to the aforementioned A coefficients. Substituting the field into Eq.(8) and using Eq.(9) one obtains the leaky-mode Purcell coefficient which is independent from arbitrary values of A^{-}_{TM} and A^{-}_{TE} . Similarly to the waveguide modes the electric field of the leaky modes with $m = \pm 1$ is radial and for TM-mode with $m = 0$ it has axial direction.

Section S3. On the directionality of emission from NW.

In experiment radiation emitted by quantum dot in vertically standing NW or NW on the substrate is collected by objective, characterized by certain numerical aperture or cone angle. Figure S5 demonstrate the ratio of radiation power collected by objective oriented along the axis NW exceeds to corresponding value for orientation of objective perpendicular to the axis of NW for radial and axial orientation of dipole (directional parameter) as a function of the cone angle for infinitely long uniform NW of the diameter 130 nm.

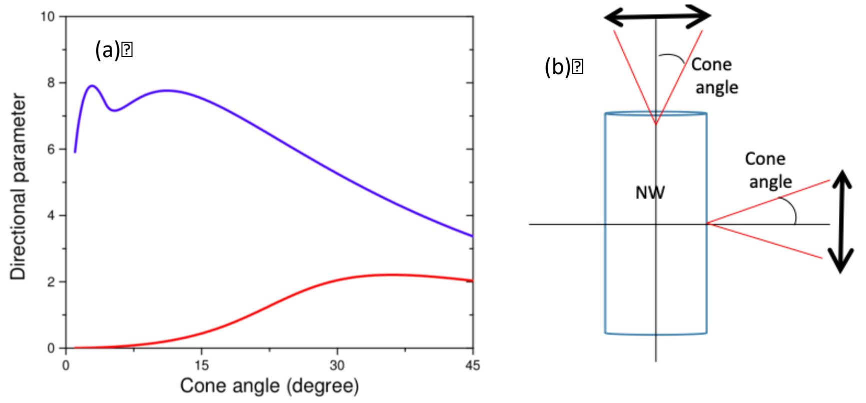

Figure S5. (**a**) Ratio of integrated emission probability inside the cone oriented along axis of NW and inside the cone oriented in perpendicular direction for axial orientation of dipole (blue) and radial orientation (red) as a function of the cone angle; (**b**) The scheme illustrating calculation of the Directional parameter.

It can be seen, that for our experimental condition, for axial orientation of the dipole, the emission probability in vertical (axial) direction is about 7 times larger, than radial direction. At the same time, for radial orientation of the dipole, directional parameter is comparable to unity.

An emission pattern of the NW can be influenced by the substrate via positive or negative interference by the wave emitted outward the substrate and the wave emitted toward the substrate and reflected back.

Figure S6 shows the distribution of the time-averaged Poynting vector around the NW, standing vertically on a Si substrate and lying on a Si substrate. As can be seen from the Figure S2, the radiation from the lying nanowire is partly taken by a substrate.

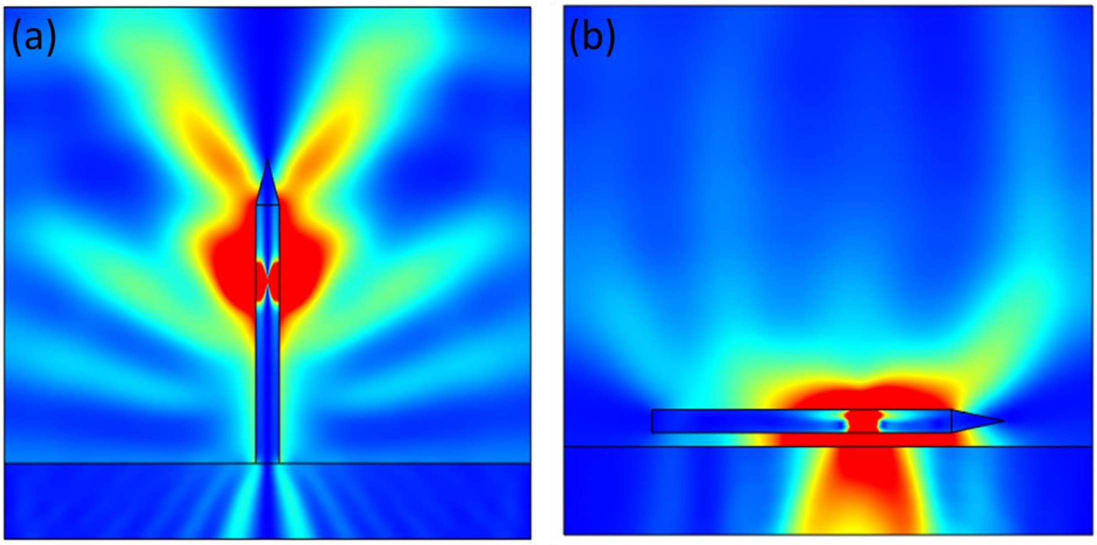

Figure S6. Distribution of the time-averaged Poynting vector around the NW, standing vertically on a Si substrate **(a)** and lying on a Si substrate **(b)**.

Emission pattern in AlGaAs nanowires with GaAs quantum dots have been studied using simulations in the “Electromagnetic Waves, Frequency Domain” module of COMSOL multiphysics software. The proportions of the nanowire were taken close to the experimentally grown NW (diameter 130 nm, height 2 μm). The refractive index for AlGaAs is n_NW=3.5 [4], for a silicon substrate is n_sub=3.7 [5]. We used the "Scattering Boundary Condition" on the boundaries of the model. In the model, we have used a free triangular mesh with the maximum cell size of 5 nm and 20 nm for NW and for the ambient media, respectively. An "Electric Point Dipole" was placed in the center of the nanowire at a distance of 1.2 μm from the base with an orientation parallel to the nanowire axis.

Qualitative analysis shows, that for lying NW total flow of radiation into substrate exceeds the flow of radiation outward the substrate by the factor of five.

Together with effect of directional emission described above, effect of substrate provides the two order of magnitude difference between observed emission intensities for standing and lying NW.

References

1. Chu, D.Y.; Ho, S.T. Spontaneous emission from excitons in cylindrical dielectric waveguides and the spontaneous-emission factor of microcavity ring lasers. *J. Opt. Soc. Am. B* **1993**, 10, 381-390. https://doi.org/10.1364/JOSAB.10.000381
2. Bleuse, J.; Claudon, J.; Creasey, M.; Malik, N.S.; Gérard, J.M.; Maksymov, I.; Hugonin, J.P.; Lalanne, Ph. Inhibition, Enhancement, and Control of Spontaneous Emission in Photonic Nanowires. *Phys. Rev. Lett.* **2011**, 106, 103601. https://doi.org/10.1103/PhysRevLett.106.103601
3. Nikolaev, V.V.; Ivanov, K.A.; Morozov, K.M.; Belonovski, A.V. Scattering matrix method for calculating spontaneous emission probability in cylindrically symmetrical structures. *Semiconductors* **2020**, 54, 765–773. https://doi.org/10.1134/S1063782620070106
4. D. E.; Studna, A.A. Dielectric functions and optical parameters of Si, Ge, GaP, GaAs, GaSb, InP, InAs, and InSb from 1.5 to 6.0 eV. *Phys. Rev. B* **1983**, 27, 985-1009. https://doi.org/10.1103/PhysRevB.27.985
5. Adachi, S. GaAs, AlAs, and AlxGa1−xAs: Material parameters for use in research and device applications. Journal of Applied *Physics* **1985**, 58, R1–R29 (1985). https://doi.org/10.1063/1.336070
